# Selenium-Enriched *Agaricus bisporus* Mushroom Protects against Increase in Gut Permeability *ex vivo* and Up-Regulates Glutathione Peroxidase 1 and 2 in Hyperthermally-Induced Oxidative Stress in Rats

**DOI:** 10.3390/nu6062478

**Published:** 2014-06-24

**Authors:** Tebo Maseko, Frank Rowland Dunshea, Kate Howell, Hyun-Jung Cho, Leni Rose Rivera, John Barton Furness, Ken Ng

**Affiliations:** 1 Department of Agriculture and Food Systems, Melbourne School of Land & Environment, Building 142, Parkville Campus, The University of Melbourne, Parkville, Victoria 3010, Australia; E-Mails: t.maseko@student.unimelb.edu.au (T.M.); fdunshea@unimelb.edu.au (F.R.D.); khowell@unimelb.edu.au (K.H.); 2 Department of Anatomy & Neuroscience, Melbourne Medical School, Building 181, Parkville Campus, The University of Melbourne, Parkville, Victoria 3010, Australia; E-Mails: hcho@unimelb.edu.au (H.-J.C.); leni.rivera@unimelb.edu.au (L.R.R.); j.furness@unimelb.edu.au (J.B.F.)

**Keywords:** Se-enriched mushroom, *Agaricus bisporus*, oxidative stress, heat stress, organic Se, gut permeability, glutathione peroxidise-1, glutathione peroxidise-2, α-tocopherol

## Abstract

Dietary effects of organic Se supplementation in the form of Se-enriched *Agaricus bisporus* mushroom on ileal mucosal permeability and antioxidant selenoenzymes status in heat induced oxidative stress in rats were evaluated. Acute heat stress (40 °C, 21% relative humidity, 90 min exposure) increased ileum baseline short circuit current (Isc; 2.40-fold) and epithelial conductance (Ge; 2.74-fold). Dietary supplementation with Se-enriched *A. bisporus* (1 µg Se/g feed) reduced (*p* < 0.05) ileum Isc and Ge during heat stress to 1.74 and 1.91 fold, respectively, indicating protection from heat stress-induced mucosal permeability increase. The expression of ileum glutathione peroxidase (GPx-) 1 and 2 mRNAs were up-regulated (*p* < 0.05) by 1.90 and 1.87-fold, respectively, for non-heat stress rats on the Se-enriched diet relative to the control. The interplay between heat stress and dietary Se is complex. For rats on the control diet, heat stress alone increased ileum expression of GPx-1 (2.33-fold) and GPx-2 (2.23-fold) relative to thermoneutral conditions. For rats on the Se-enriched diet, heat stress increased (*p* < 0.05) GPx-1 expression only. Rats on Se-enriched + α-tocopherol diet exhibited increased expression of both genes (*p* < 0.05). Thus, dietary Se-enriched *A. bisporus* protected against increase in ileum permeability and up-regulated GPx-1 and GPx-2 expression, selenoenzymes relevant to mitigating oxidative stress.

## 1. Introduction

Acute stress and other pathological conditions disrupt gastrointestinal physiology and barrier function making the gut organs vulnerable to various disorders [1,2,3,4]. The gastrointestinal mucosa is made up of the lamina propria and a covering of a single layer of epithelial cells joined together by tight junctions to create a barrier restricting the uptake of material from the lumen. The lamina propria contains immunocytes including eosinophils, neutrophils, macrophages, lymphocytes and mast cells that protect the gut against microorganisms and their toxic products [5]. However, in leaky gut induced by stress factors such as heat, the tight junction is disrupted causing gastrointestinal barrier dysfunction arising from increased epithelial permeability [6]. This leads to high infection rates and uptake of bacterial endotoxins triggering local inflammation and immune responses [5].

A “leaky” gut is characterized by elevation in epithelial ionic conductance through the paracellular pathway [2,7]. The ion conductance is normally restricted by the tight junctional complex and the relative apposition of basolateral membranes of adjacent epithelial cells that determine the volume of the surrounding aqueous column known as the lateral intercellular space. Stress factors induce a “leaky” gut by disrupting the tight junctional complex causing shedding of the epithelial layer leading to increased permeability [8]. Epithelial permeability can be determined by measuring tissue baseline short circuit current (I_sc_) and conductance (G_e_) using an Ussing chamber [8]. For example, both restraint stress and cold restraint stress have been shown to increase I_sc _and G_e _in the jejunum of Wistar-Kyoto rat using Ussing chamber measurements, and the increased tissue permeability was confirmed by the higher flux of [^3^H]-mannitol and [^51^Cr]-labelled EDTA through the tissue [9]. 

Evidence suggested that oxidative stress induced by heat or other factors is characterised by the accumulation of reactive oxygen (ROS) and nitrogen (RNS) species, and that they are significant contributing factors in the pathogenesis of gastrointestinal tract ailments such as inflammatory bowel disease, fibrosis, ulcerative colitis and colon cancer [1,10]. Selenium is an essential trace element and a micronutrient required for several physiological functions in mammals [11,12]. Dietary organic Se has been linked to beneficial biological effects and disease prevention [11,12]. The health benefits of organic Se come about through the expression of selenoamino acids containing selenoproteins and selenoenzymes that are involved in mitigating the effects of cellular oxidative stress by inactivating cellular oxidants such as ROS and RNS [13,14,15]. These included the antioxidant selenoenzymes glutathione peroxidases (GPxs) and thioredoxin reductases (TrxRs) [4,16] that play a central role in protecting cells against oxidative injury [13,17]. In mouse, supplementation with Se-enriched milk proteins and Se-yeast up-regulated the expression of gut antioxidant selenoproteins, enhancing the capacity for cell protection from oxidative damage [18].

We have previously cultivated Se-enriched *Agaricus bisporus* mushroom by irrigating growth compost with sodium selenite and chemically characterized the organic Se that is primarily made up of selenocysteine rich selenoproteins [19]. We have also demonstrated that dietary Se supplementation with the Se-enriched *A. bisporus* significantly up-regulated GPx-1 activity, and mRNA expression of GPx-1 and gastrointestinal specific GPx-2, in rat colon [20] genes linked with anti-inflammatory properties and anti-cancer function in the gastrointestinal tract [10].

In this study, we evaluated the effects of a high Se diet in the form of the Se-enriched *A. bisporus* with or without additional α-tocopherol on ileum epithelium permeability and regulation of ileum GPx-1 and GPx-2 expression in hyperthermally induced oxidative stress in rat to determine whether there is a link between dietary Se supplementation and gut function.

## 2. Materials and Methods

### 2.1. Se-Enriched and Non-Se Enriched Agaricus bisporus Mushroom

Se-enriched *A. bisporus* (button mushroom) was cultivated by irrigation of growth compost with sodium selenite solution using grower kits supplied by a commercial mushroom producer (Mushroom Exchange Pty. Ltd., Mernda, Victoria, Australia) as described elsewhere [19]. Non-Se enriched *A. bisporus* grown under normal mushroom growing conditions was also supplied by Mushroom Exchange Pty. Ltd*.* Mushroom caps were harvested, frozen at −80 °C, and then freeze-dried. The lyophilised caps were ground into fine powders using a commercial blender and stored at RT in moisture free sealable packs until required for preparation of rat feeds.

### 2.2. Diets: Preparation of Control; Se-Enriched; Se-Enriched + α-Tocopherol Diets

Rat feed was prepared in the form of pellets by Specialty Feeds Inc. (Glen Forrest, Western Australia). Three diet types including the control were prepared. Control mushroom feed pellets were prepared by supplementing a low Se rodent feed formulation AIN 93G (10 kg; composition detailed in [18]) with 20 g control (non-Se enriched) mushroom caps containing 2.22 µg Se/g dried caps to give a final Se content of 0.12 µg Se/g feed in the control diet (Diet 1). Se-enriched mushroom feed pellets were prepared by supplementing the low Se rodent feed formulation AIN 93G (10 kg) with 142 g of Se-enriched mushroom caps containing 62.20 µg Se/g dried caps to give a final Se content of 1 µg Se/g feed in Se-enriched feed (Diet 2). The Se-enriched mushroom feed pellets supplemented with α-tocopherol (0.03% w/w) were similarly prepared as diet 2 but with the addition of α-tocopherol, to give a final Se and α-tocopherol contents of 1 µg Se + 300 µg α-tocopherol/g feed (Diet 3).

### 2.3. Animals

A total of 48 nine week-old Sprague Dawley male rats weighing 302*–*426 g obtained from a colony without known adventitious viruses, mycoplasma, enteric pathogenic bacteria and parasites were purchased from the Monash University Animal Services, Melbourne, Australia. The animal experiment protocols were approved by the Melbourne School of Land & Environment Research Animal Ethics Committee, University of Melbourne (ethics approval No. 1312820.1). Rats were randomly divided into three experimental groups and housed two per cage. The rats were housed in an air-conditioned, temperature controlled animal facility with a 12 h light-dark cycle at 21 °C. Rats were given free access to food and water at all times.

### 2.4. Animal Feeding

Rats were randomly assigned to three experimental diets: diet 1, control (0.12 µg Se/g feed); diet 2, Se-enriched (1 µg Se/g feed); and diet 3, Se-enriched + α-tocopherol (1 µg Se + 300 µg α-tocopherol/g feed). All rats were at first acclimatised with free access to control diet 1 and water for one week before the animals were given their respective diet 1, diet 2 or diet 3 and water *ad libitum* for a further period of 3 weeks. Body weights of the animals were recorded at the start of the experiment and continued weekly to monitor their growth which was normal. Their food intake and behaviour were also monitored throughout. These were also normal.

### 2.5. Acute Heat Stress Protocol

After 21 days of feeding of the allocated diets, rats from each diet were randomly allocated to thermoneutral and acute heat stress treatment groups (Table 1) and their body weights determined. Thermoneutral Groups 1, 2 and 3 were exposed to an ambient temperature of 21 °C and had their feed removed but allowed free access to water for a 90 min period. Acute heat stress Groups 4, 5 and 6 were housed individually in cages, exposed to 40 °C and 21% relative humidity (RH) and also had their feed removed and allowed free access to water for a 90 min period in a temperature controlled room. Preliminary observation of rats indicated that they can tolerate 40 °C and 21% RH conditions for up to 90 min but beyond that time point distress symptoms such as hyperventilation and lethargy set in, thus the thermoneutral and acute heat stress condition was limited to 90 min exposure.

Animals from both treatments were monitored every 10 min over the 90 min treatment duration for rectal temperatures using a temperature probe (Vicks Speed-Read Digital thermometer, 10 mm × 3 mm probe) and heart rates using a stethoscope (as beats per minute: bpm). Lubricant was used to aid thermometer probe insertion into the rectum. At the end of the treatments, rats were allowed to rest for 20 min at RT before being anaesthetised and sacrificed for tissue excision.

**Table 1:** Rat treatment groups.

Treatment Groups	Treatment (Temperature, °C)	Diet
Group 1 (*n* = 8)	Thermoneutral (T_21_)	Diet 1 (Control; 0.12 μg Se/g feed)
Group 2 (*n* = 8)	Thermoneutral (T_21_)	Diet 2 (Se-enriched; 1 μg Se/g feed)
Group 3 (*n* = 8)	Thermoneutral (T_21_)	Diet 3 (Se-enriched + α-tocopherol; 1 μg Se + 0.3 μg α-tocopherol/g feed)
Group 4 (*n* = 8)	Heat stress (T_40_)	Diet 1 (Control; 0.12 μg Se/g feed)
Group 5 (*n* = 8)	Heat stress (T_40_)	Diet 2 (Se-enriched; 1 μg Se/g feed)
Group 6 (*n* = 8)	Heat stress (T_40_)	Diet 3 (Se-enriched + α-tocopherol; 1 μg Se + 0.3 μg α-tocopherol/g feed)

### 2.6. Animal Euthanasia and Ileum Tissue Excision

Animals were killed with an initial step of a single intra-peritoneal injection of ketamine and xylazine mix to anaesthetise and an overdose of the ketamine and xylazine mix as the final euthanasia step. Ileum tissue was recovered from each rat, contents flushed out, cut into 2 cm segments and was placed in 37 °C Krebs bicarbonate buffer pH 7.4, aerated with 10% CO_2_/90% O_2_, containing 25 mM NaHCO_3_, 1.2 mM CaCl_2_, 10 mM glucose, and 0.01 M nicardipine to prevent muscle contraction.

### 2.7. Ussing Chamber Analysis

The ileum was cut open along the mesenteric border to expose the mucosa and held open handling only the edges with the aid of pins. The opened ileum segments were lifted with forceps (handling edges only) and carefully without touching the mucosal side mounted on P2311 Ussing Chamber sliders with 0.3 cm^2^ aperture areas. Tissue mounts were secured in place over the slider apertures by pins around the apertures. Excess tissue was removed from around the pins, and silicone grease was applied to both the bottom and top parts of the sliders before mounting on the tissue to ensure a water tight seal. Tissue was kept moist at all times with a few drops of Krebs buffer.

Ussing sliders with tissue were inserted into two-part chambers (EasyMount Diffusion Chambers, Physiologic Instruments, Navicyte SDR Clinical Technology, 213 Eastern Valley Way, NSW 2068, Australia) that exposed 0.3 cm^2^ of serosal and mucosal surface areas to Krebs bicarbonate buffer (115 mM NaCl, 25 mM NaHCO_3_, 2.4 mM K_2_HPO_4_, 1.2 mM CaCl_2_, 1.2 MgCl_2_, 0.4 mM KH_2_PO_4_, pH 7.4) at 37 °C and gassed with carbogen (10% CO_2_, 90% O_2_). Each chamber half contained 5 mL of the Krebs bicarbonate buffer, with the serosal bath having an additional 10 mM glucose to provide an energy substrate and the mucosal bath containing an additional 10 mM mannitol to maintain osmotic balance across the mucosa on the tissues. Each chamber had a set of four electrodes (two voltage sensing and two current passing electrodes) installed on opposite sides of the tissue and connected to the amplifier through agar bridges. Each hemi-chamber was bubbled with carbogen.

A Multichannel Voltage-Current Clamp (Physiologic Instruments, model VCC MC6) linked to the chambers was used to record baseline short circuit current (I_sc_). Epithelial conductance (G_e_) (in mS/cm^2^) of ileum tissue was determined from the current/voltage relationship. Tissue mounts from all animal subjects were prepared in triplicate and allowed to equilibrate for 30 min in the chambers before measurements were made.

### 2.8. Gene Expression

#### 2.8.1. RNA Isolation

Total RNA was extracted from RNAlater^®^ solution stabilized ileum tissue (30 mg) using a commercial kit, QIAGEN RNeasy Mini Kit (QIAGEN, Victoria, Australia), and the extraction was performed in triplicates for each rat ileum. The quality (purity) and concentration of total RNA extracted was determined using NanoDrop^®^ ND-1000 UV-Vis spectrophotometer by measuring the absorbance at λ260 nm and 280 nm and determining the 260:280 absorbance ratio. Pure RNA has an A260:A280 ratio of 1.9–2.1, and the extracted RNA samples from the rats’ ileum had A260:A280 ratios between 1.96 and 2.12 (data not shown).

#### 2.8.2. cDNA Synthesis

The BIO-RAD iScript™ Select cDNA Synthesis Kit (NSW, Australia) was used to synthesise the first strand cDNA (20 µL) from 0.3 µg total RNA from each sample. The cDNA product was diluted 1:30 with nuclease-free water and used for real-time quantitative PCR.

#### 2.8.3. Real-Time Quantitative PCR

Real-time quantitative PCR of GPx-1 and GPx-2 genes was carried on an iQ™ 5 Multicolor Real-Time PCR icycler Detection System (BIORAD, NSW, Australia). Oligonucleotide primers were designed using Primer 3 software v.0.4.0 (Bioinformatics Methods and Protocols: Methods in Molecular Biology, Humana Press, Totowa, NJ, USA) based on sequences obtained from the Genbank database (Table 2). The primers were optimized and validated by conventional PCR of cDNA (data not shown). The PCR reagents for quantitative analysis were contained in the iQ SYBR Green Supermix kit from BIORAD. The PCR reactions were performed in a final volume of 20 μL containing 6 μL of diluted cDNA (1:30, v/v) and 10 µL iQ SYBR Green Supermix. Primer concentrations in the reaction mix for each gene was 250 nM for both the sense and antisense primer pairs. Nuclease free water was used to make up the final volume.

**Table 2:** Oligonucleotide primers used for real-time quantitative PCR.

Gene	Gene accession number	Primers	Primer sequence 5′–3′
*GPx1*	NM_030826	Sense	TGAGAAGTGCGAGGTGAATG
		Antisense	CGGGGACCAAATGATGTACT
*GPx2*	NM_183403	Sense	TGCCCTACCCTTATGACGAC
		Antisense	TCGATGTTGATGGTCTGGAA
*β-Actin*	NM_031144	Sense	GTCGTACCACTGGCATTGTG
		Antisense	CTCTCAGCTGTGGTGGTGAA

The cycling PCR reaction for each sample started with an initial hot start of 95 °C for 3 min as the initial denaturation step of 1 cycle, followed by 45 cycles at 95 °C for 30 s (denaturation), 60 °C for 30 s (annealing), 72 °C for 30 s (extension) and completed with a final extension step at 95 °C for 1 min. The specificity of the PCR reaction (product) was demonstrated by melting curve analysis post PCR reactions; which showed only one peak present for all PCR products of *GPx-1* and *GPx-2* test genes and the *β-actin* reference gene. A non-template (without cDNA) reaction was included with each PCR run as a negative control. The real-time quantitative PCR assay was optimised by running serial dilutions of cDNA template and using the results to generate a standard curve. The linear regression line and the coefficient of determination (*R*^2^) of the standard curve were used to evaluate whether the qPCR assay was optimised. Amplification efficiency (E) of primer pair for each gene was calculated from the slope of the standard curve. Relative gene expression for each target gene, in the test and control samples using reference *β-actin* gene as a normaliser was determined. The cycle threshold (CT) values for each target gene were normalised with the reference gene for both test and control samples, and the Pfaffl method (Biorad Real Time PCR Applications Guide: Gene Quantification) was used to calculate the relative gene expression of the target genes in the samples.

### 2.9. Statistical Analysis

The mean of triplicate determinations was used to calculate the group mean and uncertainty as standard deviation or standard error. Statistical analyses of group means by two-factor analysis of variance (ANOVA) were performed, with a subsequent multiple comparison test by Fisher’s Protected Least Significant Difference (LSD) test using GenStat (14ed). Statistical significance was defined at *p* values less than 0.05 (*p* < 0.05). Uncertainty of group means was reported to two significant figures according to the European Analytical Chemist guidelines [21]. 

## 3. Results

### 3.1. Heat Stress Physiological Parameters

#### 3.1.1. Rectal Temperatures

**Figure 1:**
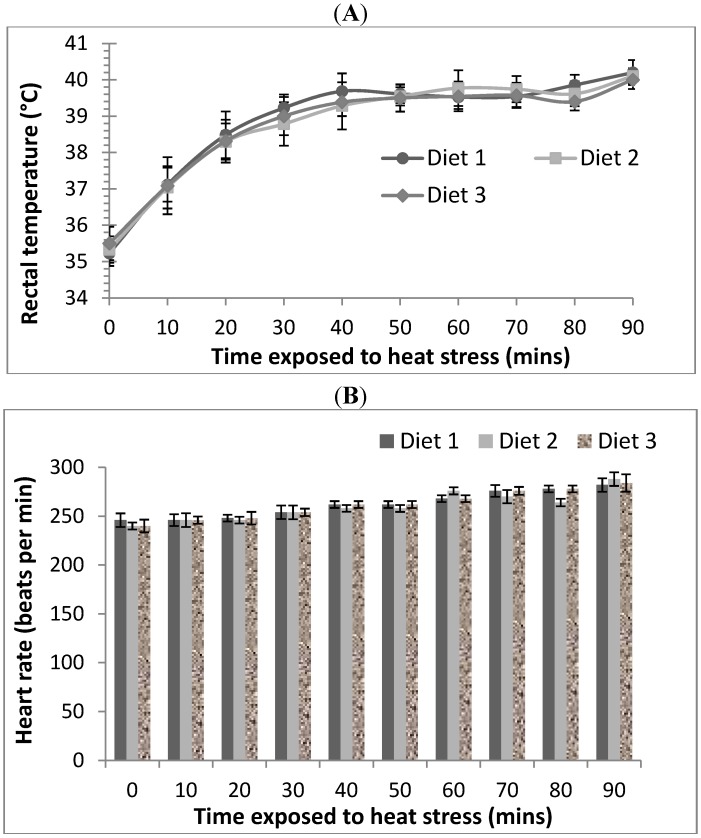
Rectal temperatures (**A**) and heart rates (**B**) of rats over time period of exposure to heat stress conditions at 40 °C and 21% RH. Mean value and standard deviation of rectal temperature of rats and heart rates from Diet 1 (*n* = 7), Diet 2 (*n* = 7) and Diet 3 (*n* = 7) were plotted.

The rectal temperatures of rats maintained under thermoneutral conditions (21 °C) were between 34.8 ± 0.3 °C and 35.9 ± 0.3 °C across all diet groups and were not changed over the 90 min duration of the treatment (data not shown). By contrast, there was an increase (*p* < 0.05) in rectal temperatures in rats exposed to the acute heat stress (40 °C, 21% RH) across all diets (Figure 1A). Rectal temperatures recorded at the start of the heat stress were 35.2 ± 0.3 °C, 35.3 ± 0.3 °C and 35.5 ± 0.4 °C for rats from Diets 1, 2 and 3 respectively. It gradually increased to 39.2 ± 0.3 °C, 38.8 ± 0.6 °C and 39.0 ± 0.5 °C for rats from Diets 1, 2 and 3, respectively, after 30 min of the heat stress condition. The rectal temperature of all heat stress rats reached 40 ± 0.4 °C at the end of the 90 min treatment.

#### 3.1.2. Heart Rates

Heart rates were also used as an indication of heat stress and were measured using a stethoscope. The heart rates of rats maintained under thermoneutral conditions were between 240 ± 3 and 246 ± 3 bpm and were not changed over the 90 min duration of the treatment (data not shown). There was a slight increase (*p* < 0.05) in heart rate in rats exposed to the acute heat stress (40 °C, 21% RH) across all diets (Figure 1B). Heart rates at the beginning of heat stress were 246 ± 6, 240 ± 3 and 240 ± 6 bpm for rats from Diets 1, 2 and 3, respectively. After 90 min of heat stress, rats recorded heart rates of 282 ± 6, 288 ± 6 and 284 ± 8 bpm for Diets 1, 2 and 3, respectively.

### 3.2. Ileum Mucosa Permeability Parameters

#### 3.2.1. Baseline Short Circuit Current (Isc)

**Figure 2:**
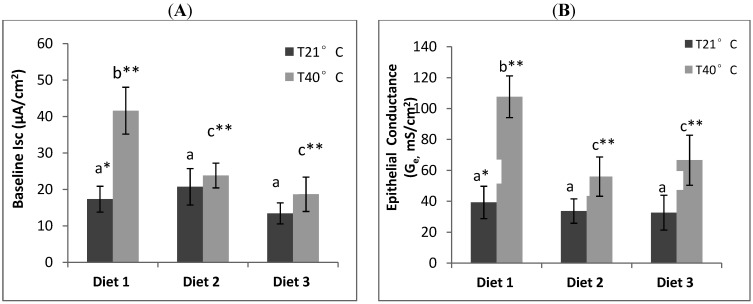
Effect of dietary Se supplementation on ileum (**A**) baseline short circuit current (Isc) and (**B**) epithelial conductance (G_e_) of non-heat stress and heat stress rats after 30 min of mounting tissue in Ussing Chamber. Number shows mean value and standard error of ileum Isc or Ge for rats on control Diet 1 (0.12 µg Se/g feed; *n* = 7), Se-enriched Diet 2 (1 µg Se/g feed; *n* = 7) and Se-enriched + α-tocopherol Diet 3 (1 µg Se/g feed + 300 µg α-tocopherol/g feed; *n* = 7) that were subjected to thermoneutral (T21°C) or heat stress (T40°C) treatments.

There were no significant differences (*p* < 0.05) in ileum I_sc_ for rats maintained under thermoneutral conditions across the three diets, at 17.3 ± 3.5, 20.7 ± 5.0 and 13.4 ± 2.8 µA/cm^2^ for Diets 1, 2 and 3, respectively (Figure 2A). However, heat stress rats displayed significantly (*p* < 0.05) higher ileum I_sc_ than non-heat stress rats across all diet groups. Heat stress rats on control Diet 1 (0.12 μg Se/g) displayed the highest ileum I_sc_ of 41.6 ± 6.4 µA/cm^2^. In addition, it was higher (*p* < 0.05) than the ileum I_sc_ of rats on Se-enriched Diet 2 (1 µg Se/g feed) and Se-enriched + α-tocopherol Diet 3 (1 µg Se + 300 µg α-tocopherol/g feed) at 23.8 ± 3.3 µA/cm^2^ and 18.7 ± 4.7 µA/cm^2^, respectively. However, there was no significant (*p* < 0.05) difference in ileum I_sc_ between heat stress rats on Diets 2 and 3.

** Mean values showing statistical significant effect relative to the control (*) (*p* < 0.05, ANOVA).

^a–c^ Mean values statistically significantly different in Fisher’s protected least significant difference test of multiple comparisons in ANOVA (*p* < 0.05).

#### 3.2.2. Epithelial Conductance (G_e_)

Current/voltage relations were determined by setting the clamp voltage at values in the range 1*–*5 mV. The data was used to calculate G_e_ (mS/cm^2^) (Figure 2B). There was no significant (*p* < 0.05) difference in ileum G_e_ for rats maintained under thermoneutral conditions across the three diets, which were 39 ± 10, 33.7 ± 7.9 and 32 ± 11 mS/cm^2^ for control diet 1, Se-enriched Diet 2 and Se-enriched + α-tocopherol Diet 3, respectively. Ileum G_e_ was significantly (*p* < 0.05) increased in heat stress rats compared to non-heat stress rats. Heat stress rats on Diet 1 displayed the highest ileum G_e_ of 107 ± 13 mS/cm^2^, and was significantly (*p* < 0.05) higher than the ileum G_e_ of heat stress rats on Diet 2 at 56 ± 12 mS/cm^2^ and Diet 3 at 66 ± 16 mS/cm^2^. However, there was no significant (*p* < 0.05) difference in ileum G_e_ between Diets 2 and 3.

### 3.3. Glutathione Peroxidase mRNA Expression

#### 3.3.1. GPx-1 mRNA Expression

**Figure 3:**
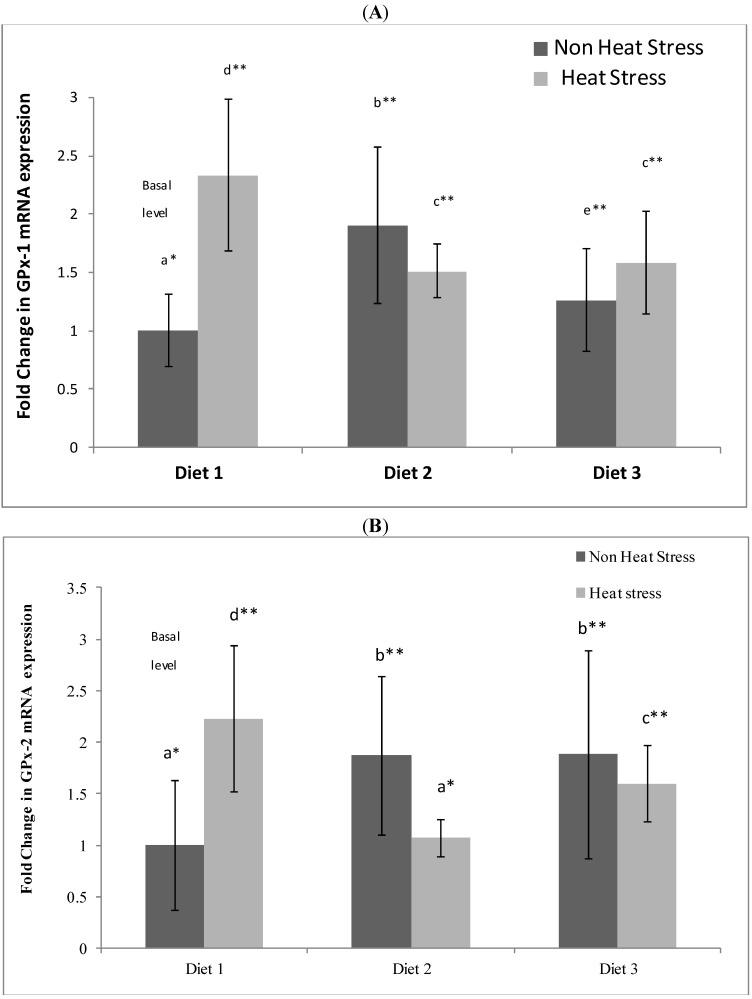
Effect of dietary Se supplementation on ileum (**A**) glutathione peroxidase-1 (GPx-1) and (**B**) glutathione peroxidase-2 (GPx-2) mRNA expression. mRNA levels were measured in triplicate ileum tissues excised from the ileum of each rat fed with control diet 1 (0.12 µg Se/g feed), Se-enriched Diet 2 (1 µg Se/g feed) or Se-enriched + α-tocopherol Diet 3 (1 µg Se/g feed + 300 µg α-tocopherol/g feed), and expression levels normalized against the β-Actin reference gene. Fold change in GPx-1 and Gpx-2 mRNA levels was calculated relative to a basal level from non-heat stress rats on control Diet 1, which was set at an arbitrary expression level of 1. Number shows mean value and standard error for rats on Diet 1 (*n* = 7), Diet 2 (*n* = 7) and Diet 3 (*n* = 7) that were subjected to thermoneutral (21 °C) or heat stress (40 °C) treatments.

There was a significant (*p* < 0.05) increase in GPx-1 mRNA expression in the ileum for rats maintained under thermoneutral conditions and fed the Se-enriched Diet 2 (1.90 ± 0.67-fold increase) and Se-enriched + α-tocopherol Diet 3 (1.26 ± 0.44-fold increase) compared to the basal level (Figure 3A). The GPx-1 expression level for rats maintained under thermoneutral conditions and fed the Diet 2 was significantly (*p* < 0.05) higher than those of rats maintained under the same condition and fed on Diet 3. Heat stress alone induced a significant (*p* < 0.05) increase in GPx-1 expression above basal level (2.33 ± 0.65-fold increase). However, while the heat stress treatment also significantly (*p* < 0.05) increased GPx-1 expression for rats on Diet 2 (1.51 ± 0.23-fold increase) and Diet 3 (1.58 ± 0.42-fold increase) above basal level, they were significantly (*p* < 0.05) lower than that of the heat stress rats on Diet 1. There was no significant (*p* < 0.05) difference in expression of GPx-1 between heat stress rat on Diets 2 and 3.

#### 3.3.2. GPx-2 mRNA Expression

As was observed for GPx-1, there was a significant (*p* < 0.05) increase in GPx-2 mRNA expression for rats maintained under thermoneutral conditions and fed the Se-enriched Diet 2 (1.87 ± 0.77-fold increase) and the Se-enriched + α-tocopherol Diet 3 (1.9 ± 1.0-fold increase) compared to the basal level (Figure 3B). The GPx-2 expression levels for rats maintained under thermoneutral conditions and fed the Diets 2 and 3 were not significantly (*p* < 0.05) different. As was also observed for GPx-1, heat stress alone was found to induce a significant (*p* < 0.05) increase in GPx-2 expression for rats on Diet 1 (2.23 ± 0.71-fold increase) above basal level. However, while the heat stress treatment also significantly (*p* < 0.05) increased GPx-2 expression for rats on Diet 3 (1.60 ± 0.37-fold increase), there was no significant (*p* < 0.05) increase for heat stress rats on Diet 2 (1.07 ± 0.18 fold increase) above basal level. GPx-2 expression level of heat stress rats on Diets 2 and 3 were, as was observed for GPx-1, significantly (*p* < 0.05) lower than that of the heat stress rats on Diet 1.

## 4. Discussion

Relatively few studies have evaluated the protective effects of dietary supplementation against gastrointestinal dysfunction due to oxidative stress. Fasting [2] and space restraint induced stress [9] have previously been shown to increase small intestinal permeability in rats. In our study, we showed the correlation between heat induced oxidative stress and gastrointestinal permeability in rats and that exposure to acute heat stress (40 °C, 21% RH, 90 min) alters epithelial physiological function in the small intestine (ileum). The elevation of both rectal temperature and heart rate of rats in the heat stress treatment was consistent with the rats experiencing heat stress. The intestinal physiological changes observed included (1) an increase in baseline short circuit current (I_sc_) which is a measure of active ion transport across the mucosa [2,9], and (2) an elevated epithelial conductance (G_e_) which is an indicator of gastrointestinal permeability [2,7,9].

The positive correlation between I_sc_ and G_e_ was expected as an elevated ion transport state correlates with an increased epithelial permeability [7,9,22]. Our study showed a protective effect of dietary mushroom Se supplementation against the increase in ileum permeability induced by acute heat stress. The I_sc_ and G_e_ of ileum from heat stressed rats placed on Se-enriched Diet 2 for 3 weeks prior to the heat stress treatment were reduced by about half compared to those of the heat stressed rats on control Diet 1. It clearly indicates a reduction of the heat stress induced elevated ion transport across gut epithelium in the rats. Thus, dietary mushroom Se supplementation might have protective effects against hyperthermally induced oxidative stress damage to epithelial functions.

It has been suggested that a combination of Se and α-tocopherol may offer better protection against epithelial injury and barrier disruption from induced oxidative stress [23]. However, we did not observe any enhanced protection against increased leakiness of the gut epithelium from heat stress with supplementation of the Se-enriched diet with α-tocopherol at the dosage evaluated in the study (300 µg/g feed). While heat stress rats on high Se and α-tocopherol containing Diet 3 had lower I_sc _and G_e _than heat stress rats on control Diet 1, they were not significantly different from heat stress rats on Diet 2 with mushroom Se only. The latter I_sc _and G_e_ were also lower than that of the control. It may well be that the mushroom Se was already providing maximum protection that a further effect could not be detected with the level of α-tocopherol used in the study.

In order to provide insight into the effect of heat stress on epithelial function at a molecular level, we determined the effect of the mushroom Se supplementation on rat ileum GPx-1 and GPx-2 expression. These are major antioxidant selenoenzymes in the gastrointestinal tract, with GPx-2 the more dominant enzyme and also expressed exclusively in the gastrointestinal tract tissues [10,24]. Thus, GPx-2, the more sensitive GPx isomer to changes in dietary Se levels [3], is believed to play a larger role in shielding the gastrointestinal tract from oxidative stress, offering protection from oxidative processes-linked inflammation aliments and cancers of the gut [3,24].

We observed that regulation of ileum glutathione peroxidases GPx-1 and GPx-2 was influenced differently by the three diets (Figure 3). The effects of mushroom Se and α-tocopherol dietary supplementation on non-heat stress rats GPx-1 and GPx-2 genes are clear. Rats maintained under thermoneutral conditions and fed the Se-enriched Diet 2 showed a markedly elevated expression of ileum GPx-1 and GPx-2 mRNA above the basal level (thermoneutral rats on control Diet 1). Rats maintained under thermoneutral conditions and fed the Se-enriched + α-tocopherol Diet 3 also showed an increase in GPx-1 and GPx-2 mRNA expression over the basal level. It appears that what we are seeing here is a chronic effect of Se on increasing GPx-1 and GPx-2 expression which is often observed [3,24], and that additional supplementation with α-tocopherol at a dosage of 300 µg/g did not impact on the Se effect as the magnitude of the fold increases from the two diets were similar. The mushroom Se-enriched dietary effect on rat ileum was similar to the same diet effect on rat colon, which we have recently shown to significantly up-regulate colonic GPx-1 activity and GPx-1 and GPx-2 mRNA expression above basal (control) level [20].

The relations between the Se diet, heat stress, gut permeability and GPxs level is complex. Even though the data did not reveal the roles of the GPx’s in the modulation of the heat-induced permeability increase by Se, it appears that Se induces GPx-1 and 2 gene expression but blunts the increased expression caused by heat exposure.

Heat stressed rats on the control Diet 1 showed a markedly elevated expression of GPx-1 and GPx-2 mRNA above the basal (thermoneutral) level. The adverse effect of heat stress is the over production of ROS in the body which is known to cause oxidative cellular damage [10] and reduced intestinal function and integrity [1,3]. Heat stress appears to produce an acute effect on the GPx-1 and GPx-2 expression as the animal attempts to counter the heat induced oxidative stress. 

Although heat stressed rats on the Se-enriched Diet 2 also showed an up-regulation of GPx-1 relative to basal level, the level was significantly lower than that of the heat stressed rats on control Diet 1. In addition, there was no observed up-regulation of GPx-2 of heat stressed rats on Diet 2 above the basal level. While it is recognised that genes expression levels might not be reflective of enzyme activity [3,10], further studies would be required to clarify their relationships. Other selenoproteins that could possibly have roles in protection against heat stress were not investigated in this study.

The observed effect of α-tocopherol and heat stress on the expression of the GPxs is rather complicated in the Se-enriched dietary regime. While heat stressed rats on the Se-enriched Diet 2 increased GPx-1 expression only, heat stressed rats on Se-enriched + α-tocopherol Diet 3 exhibited increased expressions of both *GPx-1* and *GPx-2* genes when compared to the basal level. It has been reported that dietary Vitamin E supplementation affected a number of endogenous antioxidants, and that Se is more effective at influencing GPxs up-regulation in carcinogenic malathion challenged rats than α-tocopherol, the latter being more effective at increasing the activity of non-seleno antioxidants such as catalase [23]. The continued high expression of GPx-2 in rats in the presence of α-tocopherol in the diet points to the special role of the gastrointestinal specific GPx-2 in protection against oxidative stress in the intestine. However, some studies have demonstrated synergism between Se and vitamin E, for example in the genetic inactivation of tRNA[Ser]^Sec^ required for selenoprotein expression which leads to diminished cerebral selenoprotein expression and neurodegeneration in mice [25].

## 5. Conclusions

The present study provided evidence that dietary macro-fungal organic Se from Se-enriched *A. bisporus* protected the gastrointestinal tract in rats from the effects of heat induced oxidative stress, by restoring epithelial ion transport and barrier functions, and elevating the expression of GPx-1 and gastrointestinal specific GPx-2. Although α-tocopherol supplementation of the organic Se-enriched diet also displayed significant protection of the gastrointestinal tract during heat challenge, it offered no additional benefits to epithelial physiological function and tissue integrity. The relative ease in cultivating Se-enriched *A.* *bisporus* mushroom, its unique profile of bioactive Se organic species and its mitigating effects against gastrointestinal injury arising from heat stress in rats, would present the mushroom as a viable and efficient source of functional organic Se with demonstrated biological benefits.
